# miR‐143‐3p/TET1 Axis Regulates GPC1 Through DNA Methylation and Impairs the Malignant Biological Behaviour of HCC via the Hippo Signalling Pathway

**DOI:** 10.1111/jcmm.70282

**Published:** 2025-01-17

**Authors:** Yan Liu, Di Du, Xue Gu, Qing He, Bin Xiong

**Affiliations:** ^1^ Department of Interventional Treatment The Fifth People's Hospital of Chengdu Chengdu Sichuan China; ^2^ Department of Hepatobiliary Surgery The People's Hospital of Tongnan District Chongqing city Chongqing China; ^3^ Department of General Surgery Chongqing Hospital of Traditional Chinese Medicine Chongqing China

## Abstract

Hepatocellular carcinoma (HCC) is a malignant tumour that poses a serious threat to human health and places a heavy burden on individuals and society. However, the role of GPC1 in the malignant progression of HCC is unknown. In this study, we analysed the expression of GPC1 in HCC, and its association with poor patient prognosis. The effects of GPC1 on the proliferation, invasion and migration of HCC were analysed through cellular functional experiments in vitro and in vivo. Mechanistically, DNA methylation of GPC1 was analysed by DNA extraction, methylation‐specific PCR and bisulfite Sanger sequencing (BSP), and the target genes TET1 and miRNA regulating DNA methylation of GPC1 were found through the bioinformatics database. The results revealed that GPC1 was highly expressed in HCC, and its high expression was significantly associated with poor prognosis of HCC patients. Inhibiting the expression of GPC1 can inhibit the proliferation, invasion and migration of HCC cells. GPC1 was hypomethylated in HCC, and its methylation level was regulated by TET1. miR‐143‐3p can significantly regulated the expression of TET1 and affect the methylation level and protein expression of GPC1. Furthermore, GPC1 also affects the malignant biological behaviour of HCC by regulating the expression of Hippo signalling pathway. In summary, miR‐143‐3p regulates the expression of TET1, affects the expression of GPC1 through DNA methylation and regulates the malignant progression of HCC via Hippo signalling pathway.

## 1 Introduction

Hepatocellular carcinoma (HCC) is the most common type of primary liver cancer [1]. In the early stage of HCC, surgery is the main treatment, and the clinical symptoms are not very different. The early symptoms of hepatocellular carcinoma are occulted and lack typical symptoms [2]. When there are obvious clinical symptoms, most of them have entered the middle and late stages of the disease, and even the symptoms of metastatic lesions are mainly the first manifestations [3]. Common middle and late symptoms include swelling pain in the liver area, hepatic jaundice and exoxia, progressive emaciation, fever and loss of appetite. If metastasis occurs, corresponding symptoms at the site of metastasis may appear [4]. The formation of HCC is caused by the long‐term synergistic effect of various tumorigenic factors, including genetic factors, living environment factors, lifestyle and work nature [5]. Normal liver tissue cells lose their normal regulation of growth at the gene level, resulting in uncontrolled cell proliferation and tumour formation [6]. Therefore, it is urgent to find the key genes that regulate the malignant development of HCC.

Phosphatidylinositol proteoglycan (GPC) belongs to the heparan sulfate proteoglycans (HSPGs) family [7]. Human genome encodes GPC 1–6 protein, which can be divided into GPC1/2/4/6 and GPC3/5 according to its homology [7]. At present, GPC3 has been extensively studied in this family, and elevated serum GPC3 can serve as a potential biomarker for predicting early liver cancer [8]. The single nucleotide polymorphisms at introns rs2352028 and rs3759452 in GPC5 genomic DNA are associated with the occurrence of lung cancer in nonsmoking populations [9]. However, there is still limited research on the GPC1/2/4/6 subclass. Recent studies have reported that GPC1 is highly expressed in pancreatic cancer and that GPC1 plays an important role in the neural metastasis of pancreatic cancer [10]. Studies also have reported that GPC1 is highly expressed in HCC and significantly correlated with poor prognosis of patients [11]. However, its biological role in HCC remains unclear, and the mechanism of its high expression in HCC remains unknown.

In this study, we intended to investigate the expression of GPC1 in HCC and its biological role. At the same time, further analyse the mechanism of regulating GPC1 expression and the molecular mechanism of GPC1 affecting the malignant progression of HCC.

## 2 Materials and Methods

### 2.1 Clinical Sample Collection

Sixty‐seven HCC samples (tumour tissues and adjacent nontumour tissues) were confirmed by the Department of Hepatobiliary Surgery, The People's Hospital of Tongnan District Chongqing with surgical treatment from June 2016 to December 2017. This study was approved by the Ethics Committee of the Human Trial Ethics Committee of The People's Hospital of Tongnan District Chongqing. Written informed consent was obtained from the patients who provided the specimens.

### 2.2 Cell Culture

Human normal hepatocyte THLE2, and human HCC cell lines MHCC‐97H, MHCC‐97 L, Hep3B, Huh‐7 were purchased from Shanghai Institute of Cell Biology (Shanghai, China). The cells were cultured in high‐glucose DMEM supplemented with 10% fetal bovine serum (FBS) (Gibco, USA), and cultured in a closed incubator with constant temperature of 37°C and 5% CO_2_.

### 2.3 Cell Transfection

Short hairpin RNA (shRNA) against GPC1 (Sh‐GPC1), Short hairpin RNA (shRNA) against TET1 (Sh‐TET1), and the control vector and control shRNA were purchased from Shanghai Jima Company (Shanghai, China). miR‐143‐3p mimics (miR‐143‐3p), miR‐143‐3p inhibitors and their negative controls (miR‐NC) were supplied by RiboBio (Guangzhou, China). Transfection of cell lines was performed with Lipofectamine 3000 (Thermo Fisher Scientific).

### 2.4 qRT‐PCR Analysis

Total RNA from HCC cells and tissues were using Trizol reagent (CWBIO, China). cDNA was amplified using PrimeScript RT reagent (TaKaRa, Japan). qRT‐PCR was performed with SYBR Premix Ex Taq II (TaKaRa) using a LightCycler system (Roche). GAPDH and U6 as an internal reference. Sequences of the all primers involved are listed in Table S1.

### 2.5 Western Blotting

Total protein from HCC cells and tissues were extracted mixed solution of RIPA buffer (Beyotime Institute of Biotechnology) and PMSF (Beyotime Institute of Biotechnology) on ice for 30 min. 35 μg protein was subjected to a 12% SDS‐PAGE and transferred onto PVDF membranes. The PVDF membranes were blocked with 5% nonfat‐dried milk for 1 h. The membranes were probed at 4°C overnight with primary antibodies GPC1 (1:3000; Abcam), TET1 (1:2000; Abcam), Yap (1:2000; Abcam), p‐Yap (1:1000; Abcam), Bax (1:3000; Abcam), Bcl‐2 (1:1000; Abcam), Ki‐67 (1:2000; Abcam) and GAPDH (1:5000; Abcam). The membranes were then incubated with the appropriate secondary antibodies. GAPDH was used as an internal reference.

### 2.6 Immunohistochemistry

HCC tissue sections were rehydrated in xylene and alcohol, and then rehydrated at 37°C with 3% H_2_O_2_ for 30 min. Next, all slices were incubated with goat serum for 15 min, and then wash with TBST three times 10 min each time. Then, the sections were incubated overnight with GPC1 (1:1000; Abcam) and Ki‐67 (1:1000; Abcam) at 4°C. Next, the sections incubated with antirabbit secondary IgG antibody at 37°C for 30 min. DAB (Boster) was used for visual colour rendering of the signal.

GPC1 staining classifications were as followings: the percentage of positive cells’ range 0–4: 0, negative or < 5%; 1, 6%–25%; 2, 26%–50%; 3, 51%–75%; and 4, 76%–100%. range 0–3: 0, negative; 1, weak; 2, moderate; and 3, strong. The final staining score uses the percentage of positive cells plus the staining intensity score. Grades < 4 were defined as low GPC1 expression, while Grades ≥ 4 were defined as high GPC1 expression [12].

### 2.7 DNA Extraction and Methylation‐Specific PCR

DNA was isolated from HCC cells and tissues by using a DNA Isolation kit (Tiangen). Bisulfite conversion was performed using the EpiTect Bisulfite Kit (Qiagen Inc.). Methylation‐specific PCR (MSP) was performed with 2 μL of bisulfite‐modified DNA (100 ng/50 μL) and 48 μL of PCR mixture consisting of 10× PCR Buffer (Mg2+ free), 25 mM MgCl2, dNTP mixture (each 2.5 mM), sense primer (20 μM), antisense primer (20 μM) and Takara EpiTaq HS (5 U/μL; Takara).

### 2.8 Bisulfite Sanger Sequencing (BSP)

500 ng of genomic DNA extracted from HCC cells and tissues was bisulfite‐converted using a MethylCode Bisulfite Conversion kit (Applied Biosystems; Thermo Fisher Scientific Inc.). The GPC1 promoter was amplified via PCR with Taq DNA Polymerase (Invitrogen; Thermo Fisher Scientific Inc.). The primer sequence was designed using the Methyl Primer Express Software v1.0 (Applied Biosystems; Thermo Fisher Scientific Inc.). The PCR products were electrophoresed, purified using Spin‐X tubes and then cloned into a pUC‐T vector (both from CWbiotech). Ten single products were sequenced for each sample.

### 2.9 Immunofluorescence of Cells

Hep3B and Huh‐7 cells were seeded and cultured on coverslips. Cells were fixed with 4% paraformaldehyde and permeabilised with 0.25% Triton X‐100 solution for 20 min. Cells were washed with PBS, then blocked in 5% bovine serum albumin for 1 h at room temperature. Coverslips were incubated with TET1 (1:500; Abcam, USA) and GPC1 (1:500; Abcam, USA) antibodies overnight at 4°C. After washing with PBS, cells were incubated with appropriate secondary antibody and 4′,6‐diamidino‐2‐phenylindole (DAPI).

### 2.10 Public Data Acquisition and Analysis

GPC1 and TET1 expression in HCC patients were UALCAN (http://ualcan.path.uab.edu/cgi‐bin/ualcan‐res.pl). The prognostic significance of GPC1 and TET1 was analysed using GEPIA (http://gepia.cancer‐pku.cn/index.html).

### 2.11 Dual‐Luciferase Reporter Assay

The wild‐type (WT) TET1‐3" UTR and mutant (MUT) TET1‐3" UTR oligonucleotides containing the putative binding site of miR‐143‐3p were cloned into the firefly luciferase‐expressing pMIR‐REPORT vector (Obio Technology Corp, USA). These constructs were cotransfected with miR‐143‐3p mimics, miRNA‐negative control mimics, miR‐143‐3p inhibitor and miRNA‐NC inhibitor into HCC cells. After 72 h of transfection, luciferase activity was determined using the Dual‐Luciferase Reporter Assay kit (Promega Corporation). The ratio of Renilla luciferase activity to firefly luciferase activity was calculated.

### 2.12 miRNA Prediction

TargetScan (http://www.targetscan.org), Oncomir (http://www.oncomir.org/) and miRWalk (http://mirwalk.umm.uni‐heidelberg.de/) were used to predict miRNA targets and conserved sites bound by TET1.

### 2.13 5‐Ethynyl‐2′‐Deoxyuridine (EdU) Assay

After transfection, Hep3B and Huh‐7 cells were seeded into 96‐well plates and cultured for 24 h. Then, EdU (50 μM) for 2 h at 37°C, and permeabilised with 0.5% Triton X‐100 solution for 10 min at room temperature. Cell nuclei were stained by adding 1× Hoechst 33342 (100 μL) for 30 min. Cell proliferation was analysed using the mean number cells in three fields for each sample.

### 2.14 CCK‐8 Assay

100 μL of Hep3B and Huh‐7 cells cell suspension (1 × 10^3^ cells) was added in each well of 96‐well plates. 10 μL of CCK‐8 solution (Solarbio, Beijing, China) was added to each well. The 96‐well plates was added into the cell incubator and incubated for 1 h. Finally, the absorbance of each well at 450 nm was detected.

### 2.15 Cell Migration and Invasion Assays

After transfection, Hep3B and Huh‐7 were seeded onto the upper chamber. Transwell chambers (Corning Costar, Cambridge, MA, USA) were used to detect cell migration and invasion. For the invasion assay, the inserts were precoated with Matrigel (1 mg/mL), and 500 ul of high‐glucose DMEM containing 10% FBS was added to the matched lower chamber. After incubation for 48 h, the transwell chambers were fixed in methanol and stained with 0.1% crystal violet.

### 2.16 Immunohistochemistry

Tissue sections were rehydrated in xylene and alcohol followed by 3% H_2_O_2_ for 30 min at 37°C. All sections were incubated for 15 min with goat serum to block nonspecific binding, followed by incubation with Ki‐67 (1: 5000; Abcam) at 4°C overnight. Then, the sections incubated with antirabbit secondary IgG antibody at 37°C for 30 min. The visualisation signal was achieved using diaminobenzidine (DAB, Boster).

### 2.17 In Vivo Experiments

Twenty male BALB/c nude mice (5‐weeks‐old) were purchased from the Shanghai Experimental Animal Center (Shanghai, China). Hep3B and Huh‐7 cells (1 × 10^6^ cells/mL) were subcutaneously injected into the flank of each nude mouse. Tumour volume calculated formula was as follows: volume = (width^2^ × length)/2.

### 2.18 Statistical Analyses

A paired Student's *t*‐test was used to analyse the significant difference in GPC1and TET1 expressions in HCC. Analysis of variance (ANOVA) was used for multiple group comparisons. Tukey's test was used as the post hoc test after ANOVA. The correlation between TET1 and GPC1 expressions was evaluated using Spearman's correlation analysis. The threshold for statistical significance was *p* < 0.05. All data are presented as the means ± SD. Statistical data were analysed using the SPSS 20.0 software (SPSS Inc., Chicago, IL, USA) or GraphPad Prism version 6.0 (CA, USA).

## 3 Results

### 3.1 High Expression of GPC1 in HCC and Predicts Poor Prognosis

**FIGURE 1:**
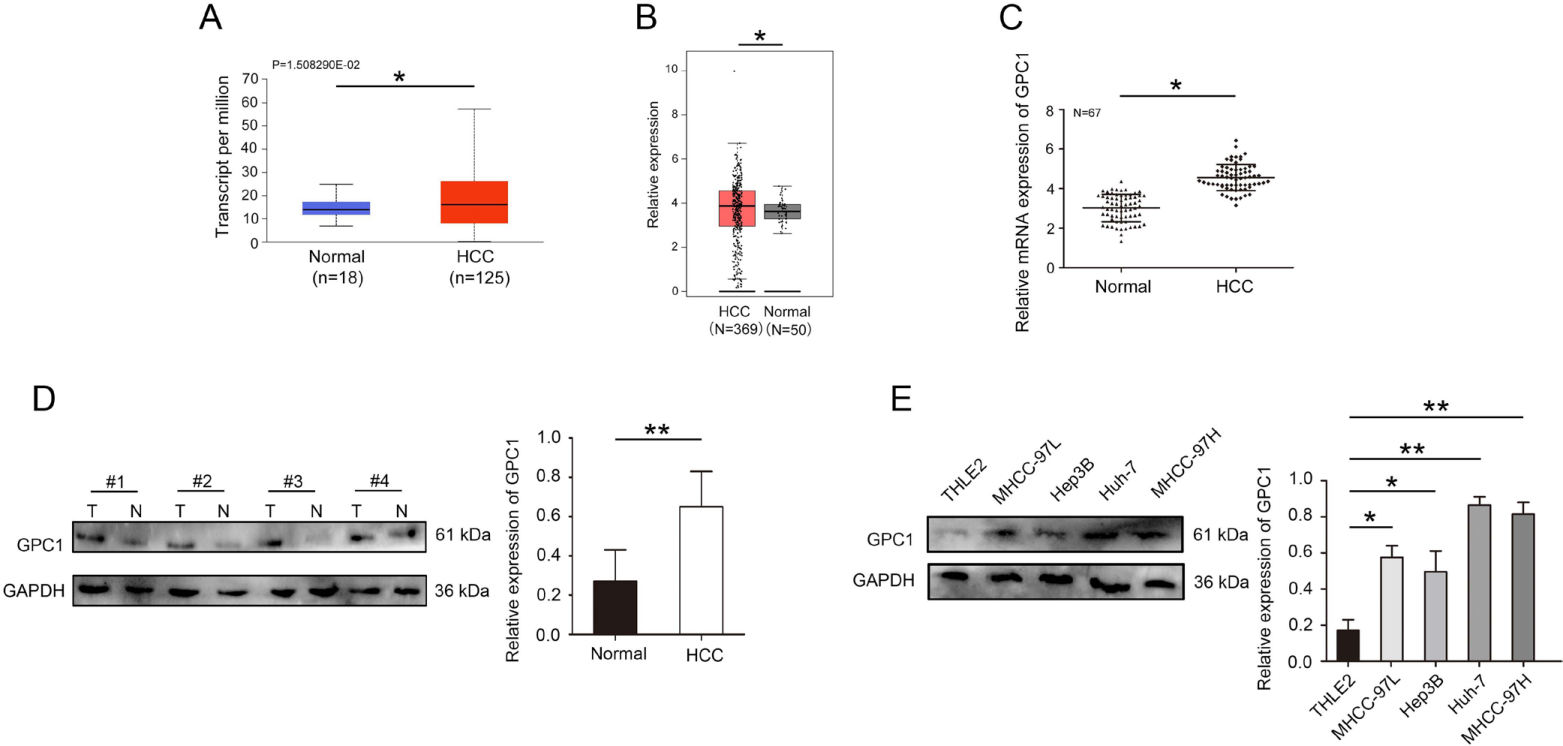
GPC1 expression was upregulated in HCC. (A) The expression of GPC1 was analysed in UALCAN database. (B) The expression of GPC1 was analysed in GEPIA database. (C) qRT‐PCR determined GPC1 expression in HCC tissues. (D) Western Blot determined GPC1 expression in HCC tissues. (E) Western Blot determined GPC1 expression in HCC cell lines. *p < 0.05, **p < 0.01.

**FIGURE 2:**
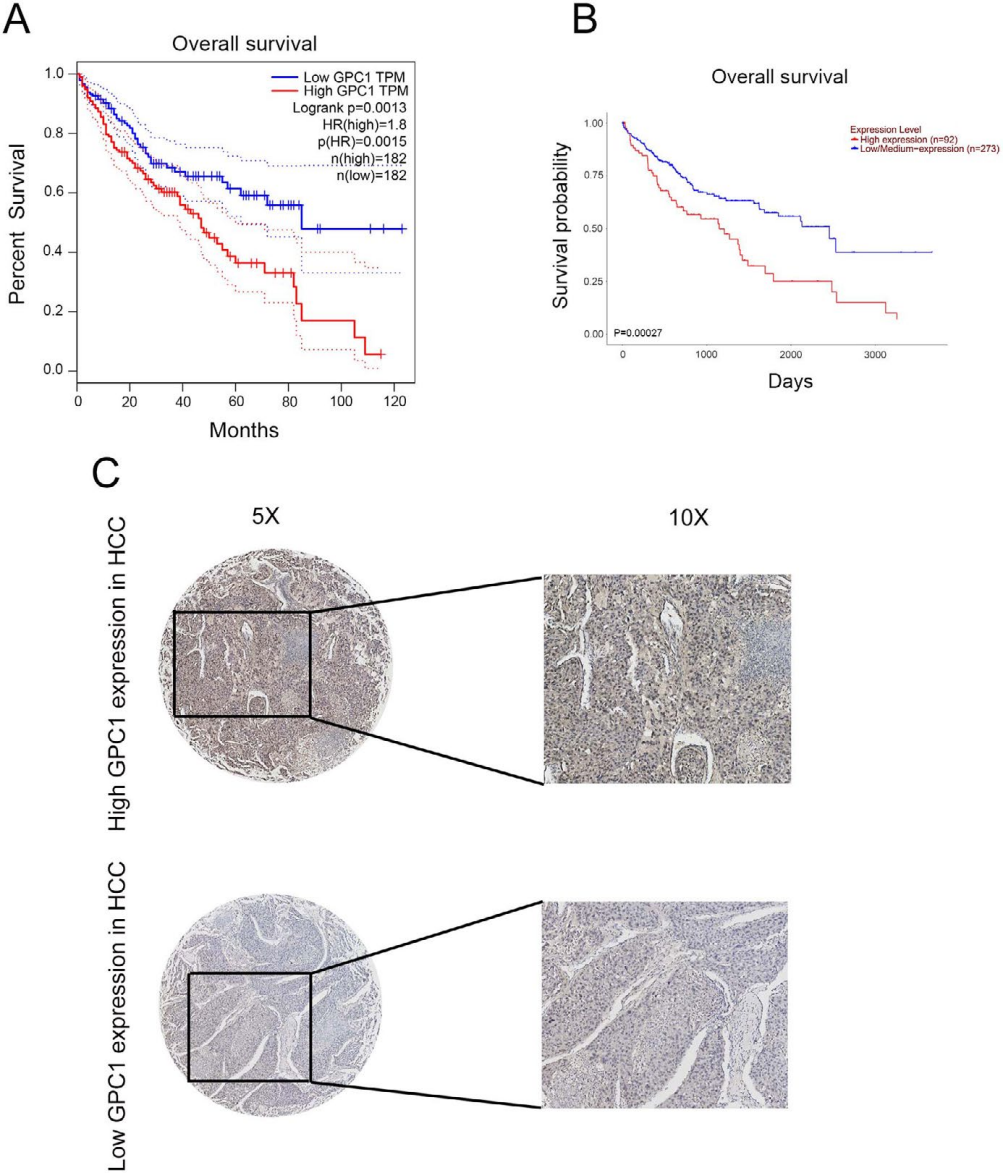
Relationship between TET1 expression and prognosis in HCC. (A) Association between GPC1 and OS in HCC patients in UALCAN database. (B) Association between GPC1 and OS in HCC patients in GEPIA database. (C) Immunohistochemistry determined GPC1 expression in HCC tissues.

Bioinformatics database data indicate that GPC1 was highly expressed in HCC (Figure 1A,B). As shown in Figure 1C, GPC1 was highly expressed in HCC tumour tissues, which was verified by qRT‐PCR. In addition, the western blot results also confirmed that GPC1 was significantly upregulated in HCC tissues and lines (Figure 1D,E). Bioinformatics data indicate that high expression of GPC1 in HCC patients has a poor prognosis (Figure 2A,B). Immunohistochemistry results also confirmed that GPC1 was significantly upregulated in HCC tissues (Figure 2C). Association between GPC1 expression and clinicopathological characteristics of HCC was investigated. GPC1 expression was significantly associated with tumour size (*p* = 0.005) and TNM stage (*p* = 0.029) (Table 1).

**TABLE 1:** Correlations between GPC1 and clinicopathological features of HCC.

Variables	Cases	GPC1 expression		*p*
		High (*n* = 46)	Low (*n* = 21)	
Age (year)				
< 50	32	21	11	0.609
≥ 50	35	25	10	
Gender				0.285
Male	32	24	8	
Female	35	22	13	
AFP (ng/mL)				0.526
≤ 20	25	16	9	
> 20	42	30	12	
HBsAg				0.498
Positive	36	26	10	
Negative	31	20	11	
Tumour size (cm)				0.005
≤ 5	31	16	15	
> 5	36	30	6	
TNM stage				0.029
I/II	31	17	14	
III/IV	36	28	7	
Tumour number				0.398
Single	37	27	10	
Multiple (≥ 2)	30	19	11	

### 3.2 Impact of GPC1 on the Malignant Biological Behaviour of HCC

**FIGURE 3:**
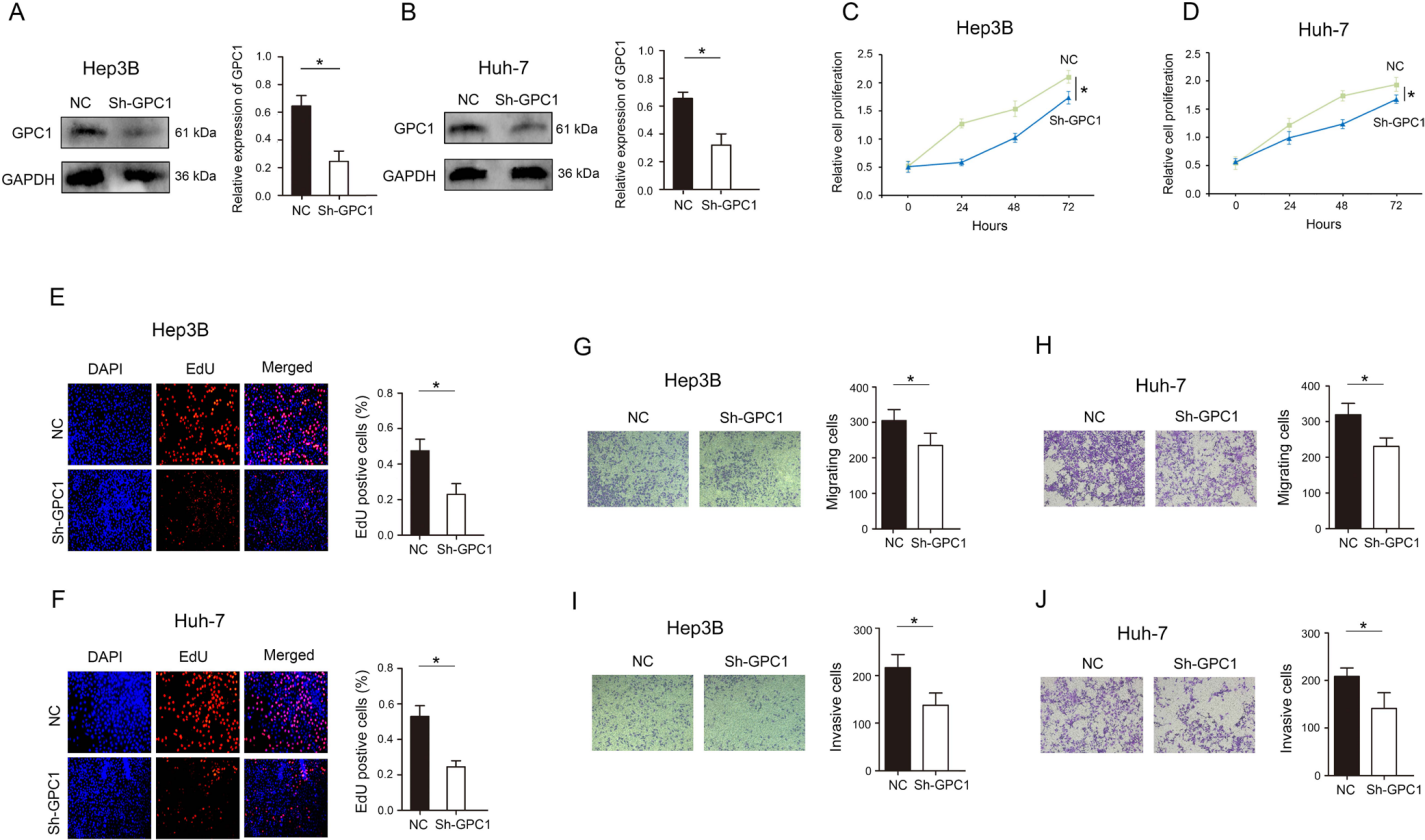
Influence of GPC1 on HCC cells. (A) Western blot assay showing the expression of GPC1 in Hep3B cells. (B) Western blot assay showing the expression of GPC1 in Huh‐7 cells. (C) CCK‐8 assay were used to detect proliferation in Hep3B cells. (D) CCK‐8 assay were used to detect proliferation in Huh‐7 cells. (E) EdU assays were used to analyse the effect of GPC1 on Hep3B cell proliferation. (F) EdU assays were used to analyse the effect of GPC1 on Huh‐7 cell proliferation. (G) Transwell assays were performed to determine the effects of GPC1 on Hep3B cell migration. (H) Transwell assays were performed to determine the effects of GPC1 on Huh‐7 cell migration. (I) Transwell assays were performed to determine the effects of GPC1 on Hep3B cell invasion. (J) Transwell assays were performed to determine the effects of GPC1 on Huh‐7 cell invasion. *p < 0.05.

First, the expression of GPC1 was effectively silenced in Hep3B and Huh‐7 (Figure 3A,B). CCK‐8 assays measured cell proliferation, indicating that decreased GPC1 expression significantly inhibited cell proliferation (Figure 3C,D). Moreover, decreased GPC1 expression significantly inhibited cell proliferation, as determined by EdU assays in Hep3B and Huh‐7 (Figure 3E,F). The mobility and invasion abilities of Hep3B and Huh‐7 with decreased GPC1 expression were obviously impaired (Figure 3G–J).

### 3.3 Impact of GPC1 on HCC Cell Proliferation In Vivo

**FIGURE 4:**
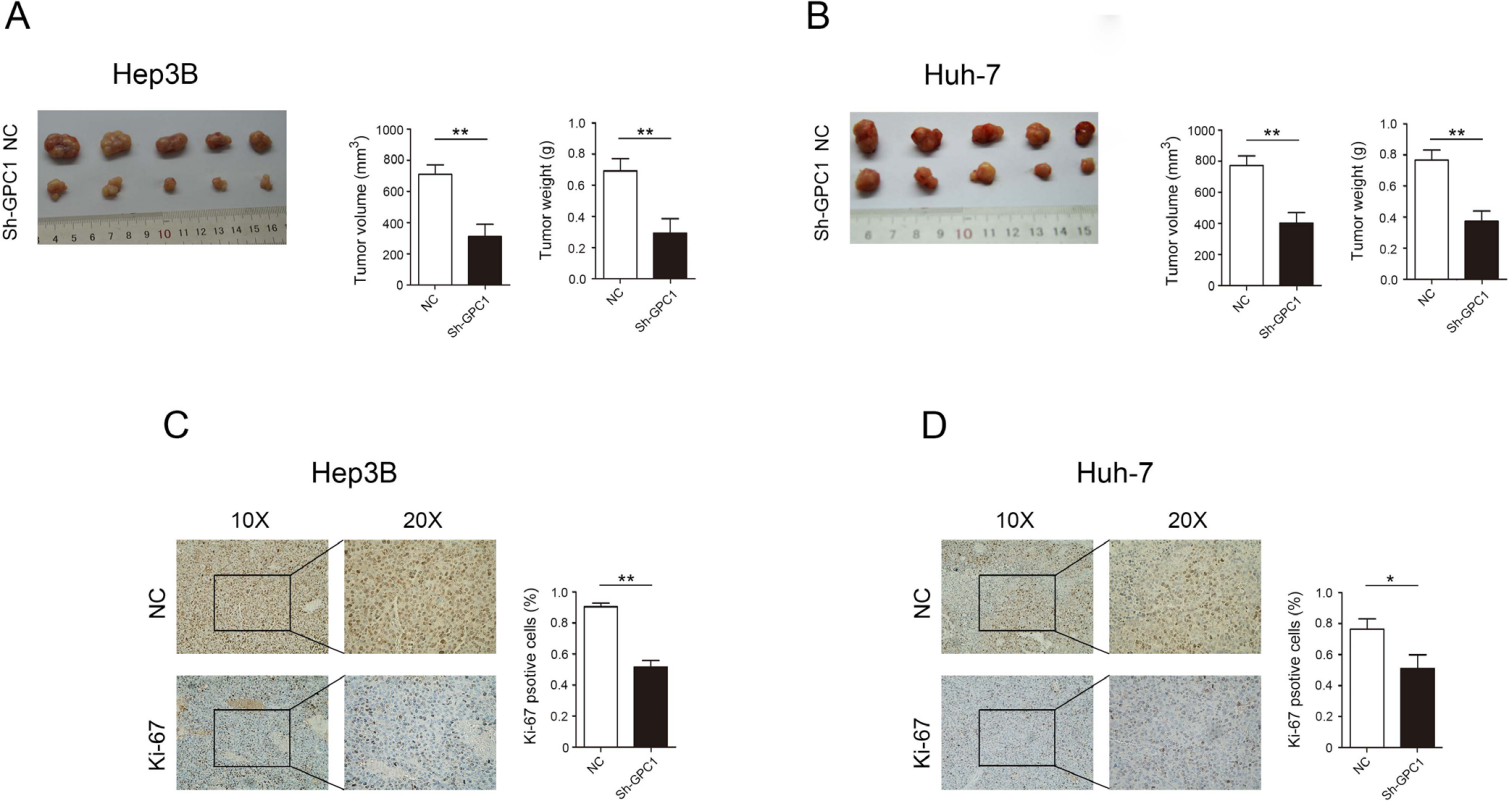
Impact of GPC1 on HCC cells in vivo. (A) Photographs of representative tumours are shown in Hep3B cells. (B) Photographs of representative tumours are shown in Huh‐7 cells. (C) The expression of Ki‐67 in Hep3B tumours. (D) The expression of Ki‐67 in Huh‐7 tumours. *p < 0.05, **p < 0.01.

In vivo HCC tumorigenesis experiment, the results showed that decrease in GPC1 expression significantly inhibits Hep3B and Huh‐7 cell proliferation (Figure 4A,B). In addition, decrease in GPC1 expression significantly inhibited the expression of Ki‐67 in Hep3B and Huh‐7 (Figure 4C,D).

### 3.4 Low DNA Methylation of GPC1 Promoter CpG Island Contributes to GPC1 High Expression in HCC

**FIGURE 5:**
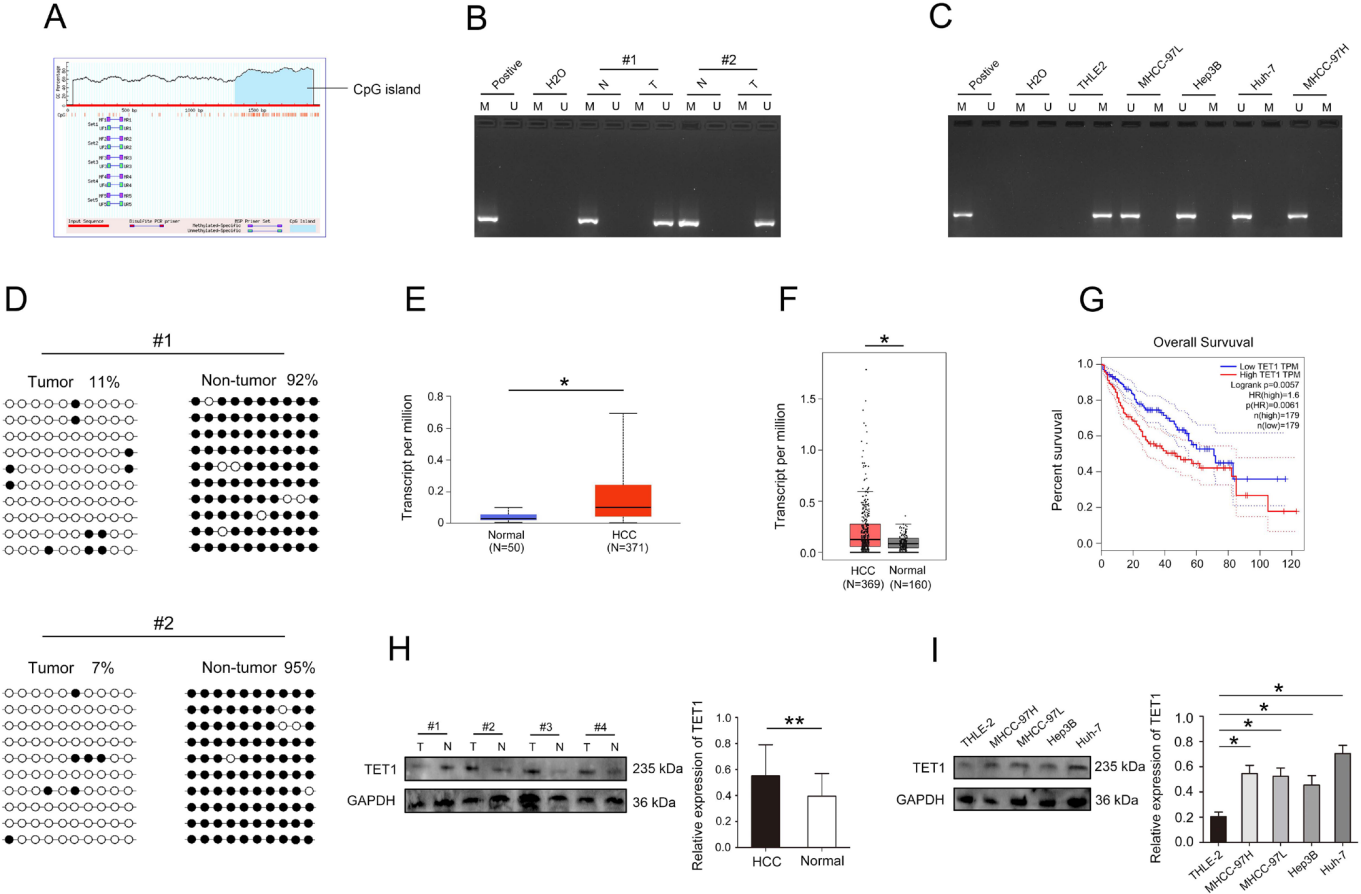
Methylation of GPC1 and aberrant expression of TET1 in HCC. (A) The CpG islands (Blue shaded area) of GPC1 promoter region. (B) The methylation status of GPC1 in HCC. (C) The methylation status of GPC1 in HCC cell lines. (D) Bisulfite sequencing analysis of GPC1 promoter methylation in HCC samlpes. (E) The expression of TET1 was analysed in UALCAN database. (F) The expression of TET1 was analysed in GEPIA database. (G) Association between TET1 and OS in HCC patients in UALCAN database. (H) Western Blot determined TET1 expression in HCC tissues. (I) Western Blot determined TET1 expression in HCC cell lines. *p < 0.05, **p < 0.01.

Biological database analysis revealed that GPC1 had CpG that could be modified by DNA methylation (Figure 5A). In addition, GPC1 promoter CpG methylation in HCC tissues was decreased (Figure 5B). Next, the methylation of GPC1 promoter CpG was downregulated in HCC cell line (Figure 5C). Moreover, BSP results showed that GPC1 promoter methylation in HCC tissues was decreased compared with adjacent nontumour tissues (Figure 5D). These results suggest that GPC1 has a low degree of DNA methylation, and DNA methylation is a dynamic regulatory process of methyltransferase and demethylase, so whether demethylase plays a key role in DNA methylation regulation of GPC1 [13]. Therefore, we further analysed the role of TET1 in DNA methylation of GPC1 [13]. Bioinformatics database data indicate that TET1 was highly expressed in HCC (Figure 5E,F), and high expression of TET1 in HCC patients has a poor prognosis (Figure 5G). In addition, the western blot results confirmed that TET1 was significantly upregulated in HCC tissues and lines (Figure 5H,I).

### 3.5 TET1 Regulates GPC1 Protein Expression Through DNA Methylation

**FIGURE 6:**
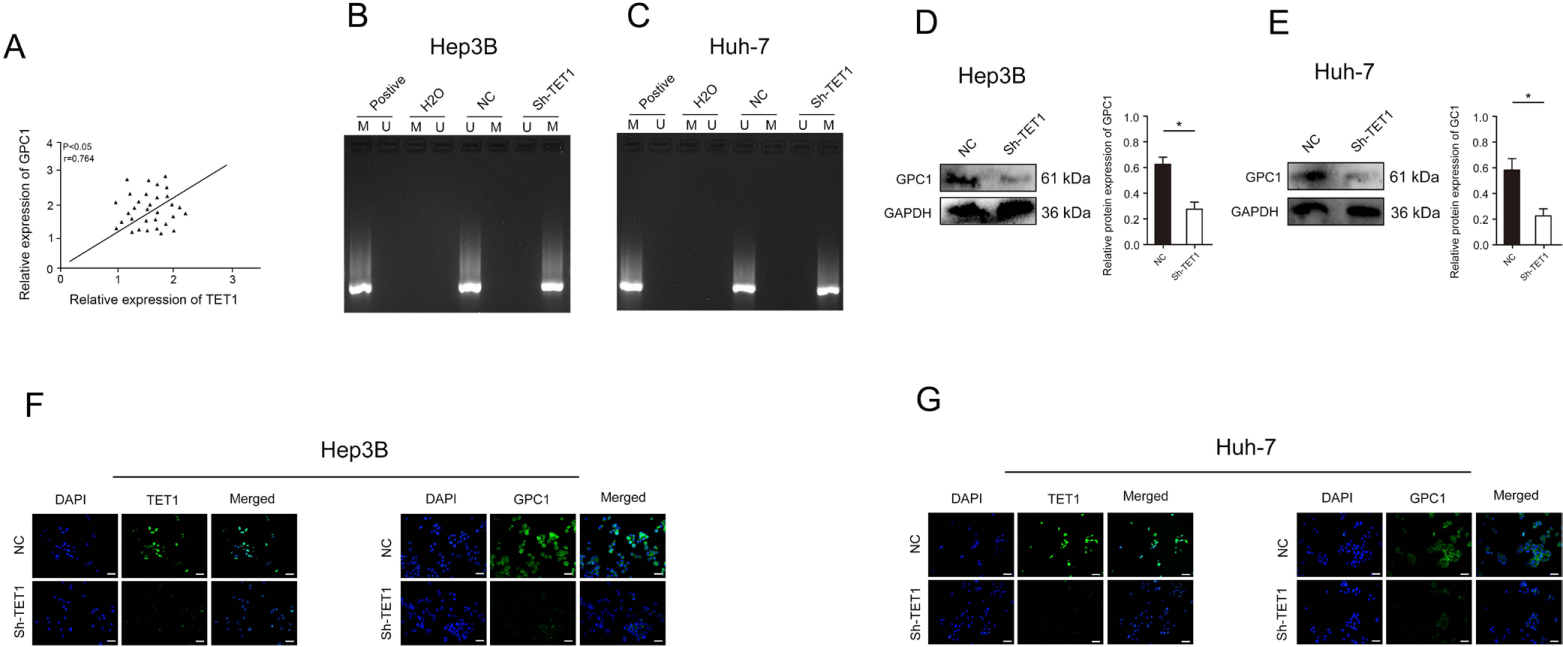
TET1 regulates GPC1 expression through DNA methylation. (A) TET1 expression was positively correlated with GPC1 expression in HCC tissues. (B) The methylation status of GPC1 after TET1 expression was decreased in Hep3B cells. (C) The methylation status of GPC1 after TET1 expression was decreased in Huh‐7 cells. (D) GPC1 protein expression after TET1 expression was decreased in Hep3B cells. (E) GPC1 protein expression after TET1 expression was decreased in Huh‐7 cells. (F) TET1 and GPC1 protein expression was detected by immunofluorescence after TDG expression was decreased in Hep3B cells. (G) TET1 and GPC1 protein expression was detected by immunofluorescence after TDG expression was decreased in Huh‐7 cells. *p < 0.05.

Statistical analysis shows a positive correlation between the expression of GPC1 and TET1 (Figure 6A). Next, decreasing the expression of TET1 increased the methylation of GPC1 (Figure 6B,C). Next, we found that the expression of GPC1 was significantly decreased after decreasing the expression of TET1 (Figure 6D,E). Immunofluorescence results of HCC cells also confirmed the above results (Figure 6F,G).

### 3.6 TET1 As a Target Gene of miR‐143‐3p

**FIGURE 7:**
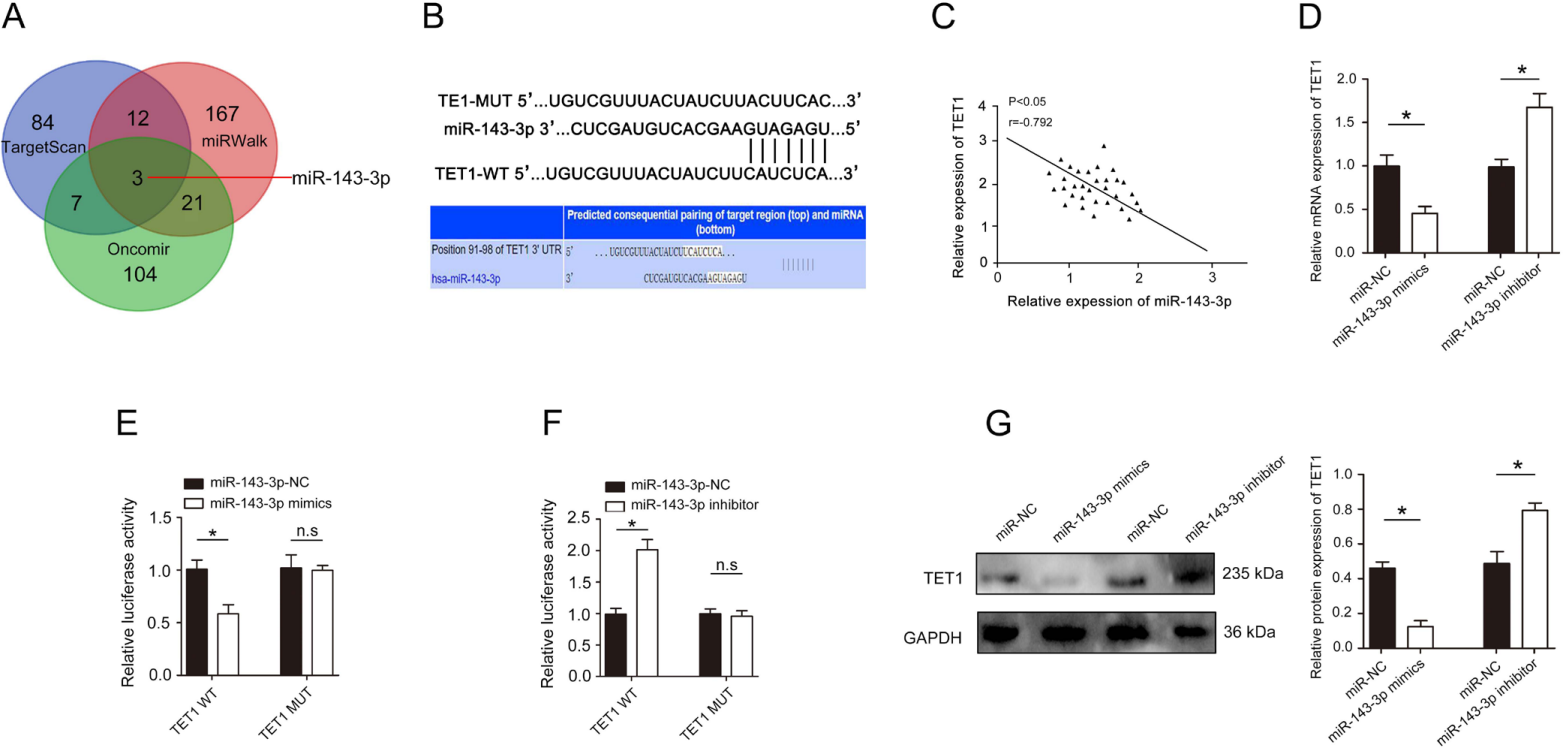
miR‐143‐3p directly targets TET1. (A) Venn diagram displaying miR‐143‐3p computationally predicted to target TET1 by TargetScan, Oncomir and miRWalk. (B) Diagrams reveal putative miR‐143‐3p binding sites and corresponding mutant sites of TET1. (C) miR‐143‐3p expression was negatively correlated with TET1 in HCC tissues. (D) The effect of miR‐143‐3p on TET1 mRNA expression. (E) Dual luciferase activity assay was performed by co‐transfection of luciferase reporter containing TET1 3' UTR or the mutant reporter with miR‐143‐3p mimics. (F) Dual luciferase activity assay was performed by cotransfection of luciferase reporter containing TET1 3' UTR or the mutant reporter with miR‐143‐3p inhibitor. (G) The effect of miR‐143‐3p on TET1 protein expression. *p < 0.05.

Through the analysis of database TargetScan, miRWalk and Oncomir, the results show that TET1 as a potential target of miR‐143‐3p (Figure 7A), and further analysis shows that miR‐143‐3p contained a potential binding site in TET1 (Figure 7B). Statistical analysis shows a negative correlation between the expression of miR‐143‐3p and TET1 (Figure 7C). qRT‐PCR assays revealed that miR‐143‐3p overexpression significantly reduced TET1 mRNA expression (Figure 7D). Dual‐luciferase reporter gene assay was employed to verify whether TET1 was a target gene of miR‐143‐3p. The results showed that cotransfection of miR‐143‐3p mimics significantly inhibited luciferase activity in cells cotransfected with Wt TET1 3'‐UTR. In contrast, inhibition was not observed in cells cotransfected with Mut TET1 3'‐UTR (Figure 7E,F). In addition, cotransfection of miR‐143‐3p inhibitor significantly increased luciferase activity in cells transfected with Wt TET1 3'‐UTR. However, the increase was not observed in cells cotransfected with Mut TET1 3'‐UTR (Figure 7F). Western blot assays revealed that miR‐143‐3p overexpression significantly reduced TET1 protein expression (Figure 7G).

### 3.7 miR‐143‐3p Ameliorates the Effect of GPC1 on HCC Progression

**FIGURE 8:**
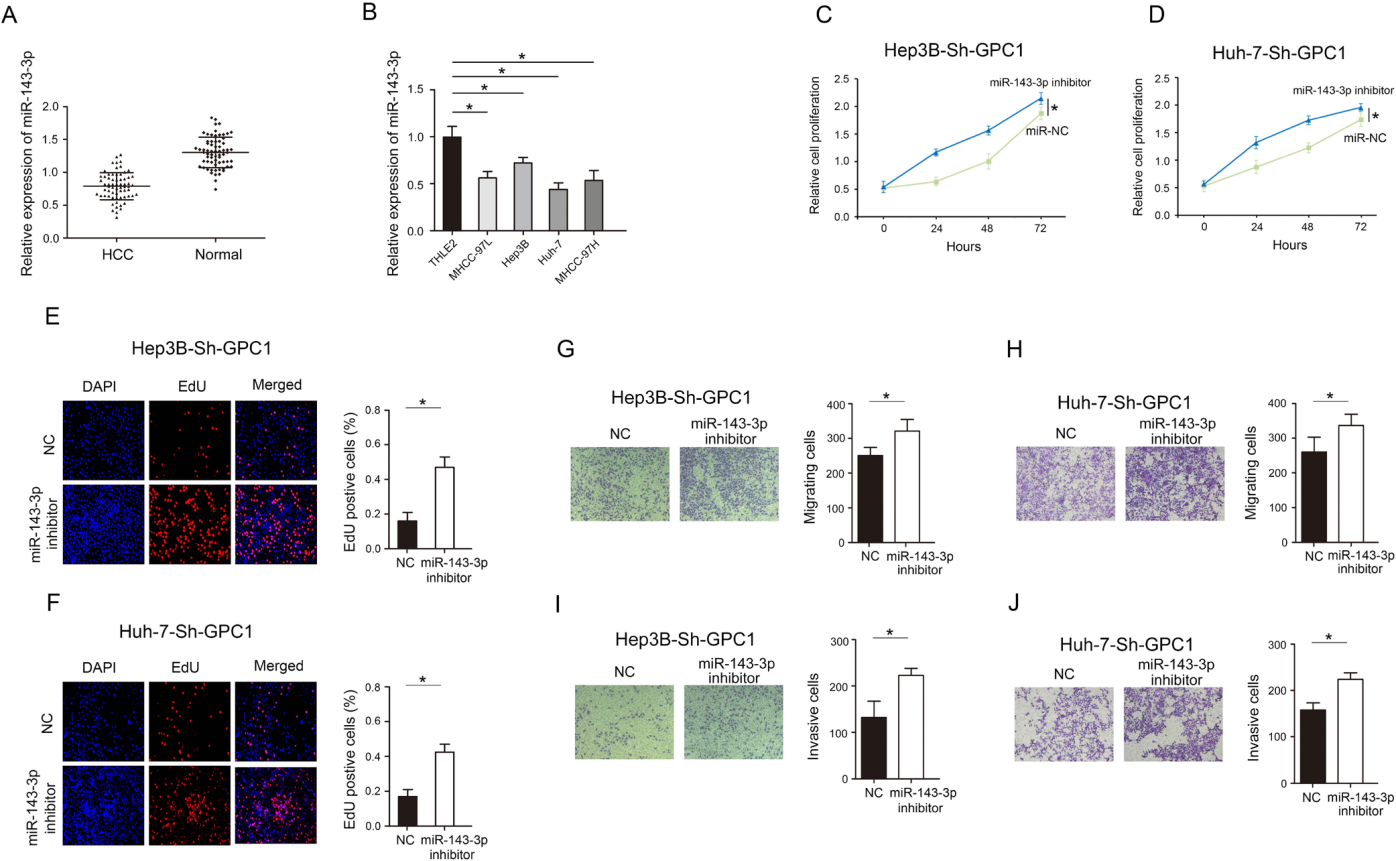
miR‐143‐3p ameliorates the effect of GPC1 on HCC progression. (A) The expression of miR‐143‐3p in HCC tissues. (B) The expression of miR‐143‐3p in HCC cell lines. (C) CCK‐8 assay were used to detect the proliferation in Hep3B‐Sh‐GPC1 cells with decreased miR‐143‐3p expression. (D) CCK‐8 assay were used to detect the proliferation in Huh‐7‐Sh‐GPC1 cells with decreased miR‐143‐3p expression. (E) Uptake capacity of EdU was analysed in Hep3B‐Sh‐GPC1 cells with decreased miR‐143‐3p. (F) Uptake capacity of EdU was analysed in Huh‐7‐Sh‐GPC1 cells with decreased miR‐143‐3p. (G) Transwell assays were performed to determine the migration of Hep3B‐Sh‐GPC1 cells with decreased miR‐143‐3p. (H) Transwell assays were performed to determine the migration of Huh‐7‐Sh‐GPC1 cells with decreased miR‐143‐3p. (I) Transwell assays were performed to determine the invasion of Hep3B‐Sh‐GPC1 cells with decreased miR‐143‐3p. (J) Transwell assays were performed to determine the invasion of Huh‐7‐Sh‐GPC1 cells with decreased miR‐143‐3p. *p < 0.05.

**FIGURE 9:**
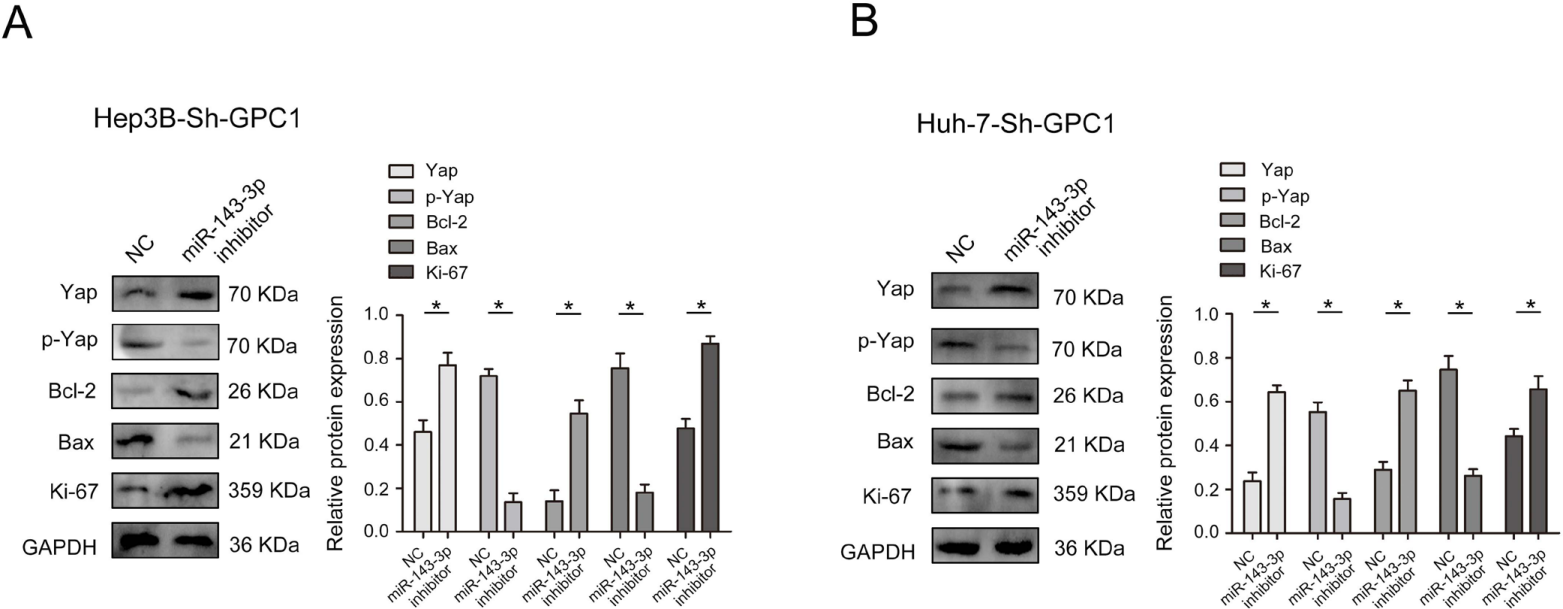
TET1 and GPC1 affect malignant progression via Hippo signalling pathway. (A) Yap, p‐Yap, Bcl‐2, Bax, and Ki67 protein expression in Hep3B‐Sh‐GPC1 cells with decreased miR‐143‐3p. (B) Yap, p‐Yap, Bcl‐2, Bax, and Ki67 protein expression in Huh‐7‐Sh‐GPC1 cells with decreased miR‐143‐3p. *p < 0.05.

As shown in Figure 8A,B, miR‐143‐3p was lowly expressed in HCC tumour tissues and cell lines, which was verified by qRT‐PCR. CCK8 and EdU assays showed that decreased of miR‐143‐3p significantly abolished the inhibitory effects of GPC1 on proliferation in HCC cells (Figure 8C–F). In addition, decrease in miR‐143‐3p significantly reduced the inhibitory effects of GPC1 on migration and invasion in HCC cells (Figure 8G–J). Hippo signalling pathway regulates cell proliferation and apoptosis to control tissue size [14], and our results demonstrated that decrease in miR‐143‐3p expression, the expression of Yap, Bcl‐2 and Ki‐67, which were inhibited by downregulation of GPC1, were restored to a certain extent, and the expression of Bax and p‐Yap, which was increased by downregulation of GPC1, was inhibited to a certain extent (Figure 9A,B).

## 4 Discussion

Epigenetics is a discipline that studies the mechanism by which genetic information of related traits is preserved and transmitted to offspring through DNA methylation, chromatin conformational change and other ways in the case of non DNA sequence changes [15, 16]. There are many epigenetic phenomena, including DNA methylation, genomic imprinting, maternal effect, gene silencing, nucleolar dominance, dormant transposon activation and RNA editing [16]. Among them, DNA methylation is the most common form of epigenetic modification. Normal methylation is necessary to maintain cell growth and metabolism, while abnormal DNA methylation can cause diseases (such as tumours), because abnormal methylation may not only allow tumour suppressor genes to be transcribed on the one hand but also lead to genomic instability on the other [17]. DNA methylation refers to the combination of a methyl group in the covalent bond at the cytosine 5 carbon position of the genome CpG dinucleotide under the action of DNA methylation transferase [18]. CpG island in the human genome, which is about 100–1000 bp in size and rich in CpG dinucleotides, is always in an unmethylated state and is associated with 56% of the human genome coding genes [18]. Human genome sequence sketch analysis results show that the human genome has about 28,890 CpG island, and most chromosomes have 5–15 CpG island per 1 Mb, with an average of 10.5 CpG island per Mb. The number of CpG island has a good correlation with gene density [18]. Due to the close relationship between DNA methylation and human development and tumour diseases, especially the inactivation of tumour suppressor gene transcription caused by CpG island methylation, DNA methylation has become an important research content of epigenetics and epigenomics [19].

DNA demethylation can be achieved through passive demethylation of DNA: Due to the lack of methylation maintenance enzymes, the methylated cytosine is diluted in the genome, or through active demethylation of DNA: 5mC is oxidised by the 10–11 translocation (TET) enzyme to an oxidised derivative of 5mC [20]. DNA demethylases mainly refer to the 10–11 translocation protein (TET) family, including TET1, TET2 and TET3, and are important enzymes that regulate DNA methylation and demethylation [21]. TET1, as a member of the TET protein family, is a type of α‐ketoglutarate and Fe2^+^dependent dioxygenases can convert 5‐methylcytosine (5mC) to 5‐hydroxymethylcytosine (5hmC), thereby initiating the DNA demethylation process [22]. Research has shown that TET1 is mainly expressed abnormally in solid tumours and can regulate the expression of oncogenes through DNA demethylation, while TET2 and TET3 play a role in regulating oncogenes and tumour suppressor genes in nonsolid tumours [23, 24]. In breast cancer, TET1 is regulated by miR‐27a‐3p. At the same time, TET1 affects the expression of ADCY6 by removing DNA methylation, thereby regulating the malignant progression of breast cancer [25]. PRMT5 binds to the transcription factors Snail and NuRD complexes to form transcriptional inhibitory complexes, regulating TET1 and E‐cadherin methylation and deacetylation to promote invasion and metastasis of cervical cancer [26]. In HCC, the expression of TET1 is significantly higher in tumour samples than in normal tissues [27]. Compared with early (I + II) and graded (G1 + G2) HCC patients, late (III + IV) and graded (G3 + G4) HCC patients have higher TET1 expression [3]. HCC patients with high expression of TET1 have poorer prognosis than those with low expression of TET1 [27]. Our results are consistent with previous studies. More importantly, we found that TET1 can significantly regulate the expression of GPC1 and regulate the proliferation, migration and invasion of HCC via DNA methylation.

MicroRNAs (miRNAs) are a class of endogenous noncoding RNA with regulatory function found in eukaryotes, with the size of about 20–25 nucleotides [28]. Mature miRNAs are produced by a series of nuclease shearing processes of longer primary transcripts, and then assembled into RNA‐induced silencing complexes [29]. They recognise target mRNA by base complementary pairing, and guide the silencing complexes to degrade target mRNA or inhibit target mRNA translation according to the degree of complementarity [30]. In recent years, it has been found that miRNAs are closely related to the occurrence and development of tumours [31]. Compared with normal tissue, the expression profile of miRNA in tumour tissue undergoes significant changes, and miRNA interacts with traditional tumour suppressor and oncogenes, playing a ‘dual role’ in the occurrence and development of tumours [32]. MiRNA can possess the function of oncogenes and downregulate the expression of tumour suppressor genes, providing favourable conditions for tumour proliferation, metastasis and infiltration, and can also have the function of tumour suppressor genes, downregulate the expression of oncogenes and make beneficial contributions to tumour apoptosis, differentiation and treatment [33]. In brain metastasis of lung cancer, m6A methyltransferase METTL3 can increase the splicing of precursor miR‐143‐3p to promote the formation of mature miR‐143‐3p. In addition, miR‐143‐3p regulates the expression of downstream VASH1 and mediates the depolymerisation of tubulin to increase cell motility [34]. MiR‐143‐3p targeting integrin α 6 (ITGA6) and with SH3 domain, anchor protein repeat sequence and PH domain 3 (ASAP3), and promote the occurrence and development of colorectal cancer by regulating the expression of both proteins [35]. In HCC, SOX2‐OT binds to miR‐143‐3p to form a complex that promotes MSI2 expression and regulates the malignant progression of HCC through downstream signalling pathways [36]. Here, we also observed that miR‐143‐3p binds to TET1 and negatively regulates its expression, affecting its DNA demethylation function and promoting the expression of CPG1.

In conclusion, our results demonstrated that GPC1 is highly expressed in HCC, and its high expression is significantly correlated with poor prognosis of HCC patients. Mechanistically, GPC1 is regulated by DNA demethylation of TET1. miR‐143‐3p regulates the expression of TET1 and affects the malignant biological processes of HCC via Hippo signalling pathway.

## Author Contributions

**Yan Liu:** conceptualization (equal), data curation (equal), funding acquisition (equal), project administration (equal), validation (equal), visualization (equal), writing – original draft (equal), writing – review and editing (equal). **Di Du:** data curation (equal), formal analysis (equal), investigation (equal), methodology (equal), project administration (equal), resources (equal), software (equal), supervision (equal), visualization (equal). **Xue Gu:** conceptualization (equal), data curation (equal), formal analysis (equal), project administration (equal), resources (equal), software (equal), validation (equal). **Qing He:** conceptualization (equal), investigation (equal), methodology (equal), project administration (equal), resources (equal), software (equal), visualization (equal). **Bin Xiong:** conceptualization (equal), investigation (equal), writing – original draft (equal), writing – review and editing (equal).

## Ethics Statement

The study protocol was reviewed and approved by the institutional review board of The People's Hospital of Tongnan District Chongqing City. All the procedures were performed in accordance with the Declaration of Helsinki and relevant policies in China. The patients/participants provided their written informed consent to participate in this study.

## Consent

All authors consent to publication.

## Conflicts of Interest

The authors declare no conflicts of interest.

## Supporting information


Table S1.

## Data Availability

The data that support the findings of this study are available on request from the corresponding author. The data are not publicly available due to privacy or ethical restrictions.
