# Supplementary material for: miR‐143‐3p/TET1 Axis Regulates GPC1 Through DNA Methylation and Impairs the Malignant Biological Behaviour of HCC via the Hippo Signalling Pathway
Source: J Cell Mol Med. 2025 Jan 17;29(2):e70282. doi: 10.1111/jcmm.70282 (PMC11740985; doi:10.1111/jcmm.70282)
Supplement: Supplementary file 1 — Table S1. [file JCMM-29-e70282-s001.docx]

**Supplementary Table 1**

Sequences of the all primers

GPC1 forward: 5’- CGG CCC CGC CAT GGA GCT CC-3’

reverse: 5’- GGC AGT TAC CGC CAC CGG GG -3’

TET1 forward: 5’-TGA TGA CAG AGG TTC TTG CAC ATA AG

-3’ reverse: 5’- CAG GTT GCA CGG TCT CAG TGT T -3’

miR-143-3p forward: 5’ - : TGA GAT GAA GCA CTG TAG CTC GTC -3’

reverse: 5’- CAG TGC AGG GTC CGA GGT AT -3’

GAPDH forward: 5’- GGA GCG ACA TCC GTC CAA AAT -3’

reverse: 5’- GGC TGT TGTCAA TCT TCT CAT GG -3’

U6 forward: 5’- CTC GCT TCG GCAGCA CA -3’

reverse: 5’- AAC GCT TCA CGA ATT TGC GT -3’
